# Development of a Hybrid Cervical Spine Clearance Algorithm in Trauma: Tackling the Challenges of the NEXUS Criteria and Canadian Cervical Spine Rule Utilization in a Low-Volume Tertiary Neuroscience Unit

**DOI:** 10.7759/cureus.96748

**Published:** 2025-11-13

**Authors:** Henry E Ajah, Obiamaka O Nzekwu

**Affiliations:** 1 Neurosurgery, Imperial College Healthcare National Health Services (NHS) Trust, London, GBR; 2 Neurology, Regions Stroke and Neuroscience Hospital, Imo State, NGA

**Keywords:** cervical immobilization, cervical spine, collar, spinal trauma, trauma

## Abstract

Missed cervical spine injuries can have a devastating impact on the outcome of the trauma patient. Prolonged and unwarranted immobilization should also be avoided to reduce the risk of any potential complications. A systematic method to decide on the possibility of cervical spine injury early is therefore paramount. The Canadian C-spine (cervical spine) rule and the NEXUS (National Emergency X-Radiography Utilization Study) criteria are internationally validated methods of resolving the cervical spine in trauma patients. Peculiarities of patient populations can limit the utility of these tools, and local trauma units often need to adapt guidelines to suit their circumstances. This article describes the development of a local guideline, a hybrid protocol for the clearance of the cervical spine in adult trauma patients, applicable to a broader patient group, its advantages, preliminary impact, and the possibility of wider adoption.

## Introduction

Spine injury is a common presentation in multiply injured trauma patients, with 55% affecting the cervical spine [1]. Recent data suggest that the prevalence of cervical spine injury in blunt trauma patients is approximately five percent [2]. Due to the potential for missed injury and the attendant catastrophic outcome, measures such as spine immobilization continue to be generously used in most emergency departments. There is a need, however, to balance this with the risks associated with unnecessary prolonged immobilization [3]. This becomes particularly challenging when a specialist opinion is needed to clear the C-spine (cervical spine), a practice that stems from the fact that no definite consensus exists for the clearance of the cervical spine following trauma in emergency departments [4].

To the Emergency Department clinician, a critical question is who needs imaging before removal of immobilization. In making this decision, resources are freed up, and attention is shifted to aggressively managing other injuries that would otherwise be challenging to tackle with an unresolved cervical spine. The presence of a head injury can make it an easier decision to make, this being the strongest independent risk factor for cervical spine injury [5]. Several decision-making tools, notably the NEXUS (National Emergency X-Radiography Utilisation Study) Criteria and the Canadian C-Spine Rule (CCR), have been developed to aid the clearance of the C-spine in trauma. These are tools that the clinician can rely upon if applicable to all patient groups, which they are not.

The Canadian C-Spine rule was published in 2001 and was intended for awake and stable patients with a Glasgow Coma Score (GCS) of 15 [6]. It provides a set of high-risk factors that will necessitate imaging: people aged 65 and above, presence of paresthesia in extremities, and dangerous mechanisms of injury. It further categorizes low-risk patients using five criteria, and in this group of patients, a range of motion assessment can be done to allow clearance of the C-spine. Low-risk factors include: a simple rear-end motor vehicle collision, sitting position in the emergency department, ambulatory at any time since injury, delayed onset of neck pain, and absence of midline cervical spine tenderness. The Canadian C-spine rule is 100% sensitive and 42.5% specific [4]. The NEXUS criteria, on the other hand, provide a simpler approach to ruling out imaging in trauma patients through its five criteria: no posterior midline cervical tenderness, no evidence of intoxication, a normal level of consciousness, no focal neurologic deficit, and no painful distracting injury [7]. The NEXUS Criteria is 99.9% sensitive and 12.9% specific [4]. While the Advanced Trauma Life Support course by the American College of Surgeons teaches these decision-making tools, a 2014 study showed only 57% of level-one trauma centers in the United States had a written cervical spine clearance protocol [8]. There are no national consensus guidelines, and local hospitals have to adapt evidence-based guidelines to the circumstances of their facilities and regions. A general gap in knowledge and inconsistent application of internationally validated guidelines (NEXUS and CCR) persists among healthcare professionals, particularly in newly established trauma units [9].

We have aimed to develop a memorable, locally applicable, and visually appealing algorithm for a neuroscience unit, which can easily be taught and relied upon to rapidly assess C-spine injury and the need for radiological imaging in trauma patients. This article describes its design, implementation, and preliminary impact on clinician confidence in decision-making in trauma.

## Technical report

The gap

The local emergency department first responders consist of junior clinicians who are experienced in the management of non-traumatic neurological patients, as this is primarily a stroke center. However, due to the strategic location of the tertiary facility along a major national highway and the availability of advanced imaging resources, trauma attendances (ambulance, referrals, and 'walk-ins') are not uncommon. An initial review of the most recent trauma admissions was conducted in the form of a morbidity and mortality review involving the junior emergency department doctors, neurosurgeons, and allied specialties. During the session, all junior doctors were required to state their knowledge of C-spine clearance algorithms in trauma and their level of confidence in managing a poly-trauma patient with suspected C-spine injury. Twelve clinicians were surveyed, and 16% (n=2) reported a working knowledge of the NEXUS criteria and the Canadian C-spine rule, out of which only 8.3% reported being confident enough to go through the criteria without needing a revision and clear the C-spine. All participating clinicians felt that a single algorithm to direct local practice in the absence of a national guideline would be beneficial.

The local algorithm

**Figure 1:**
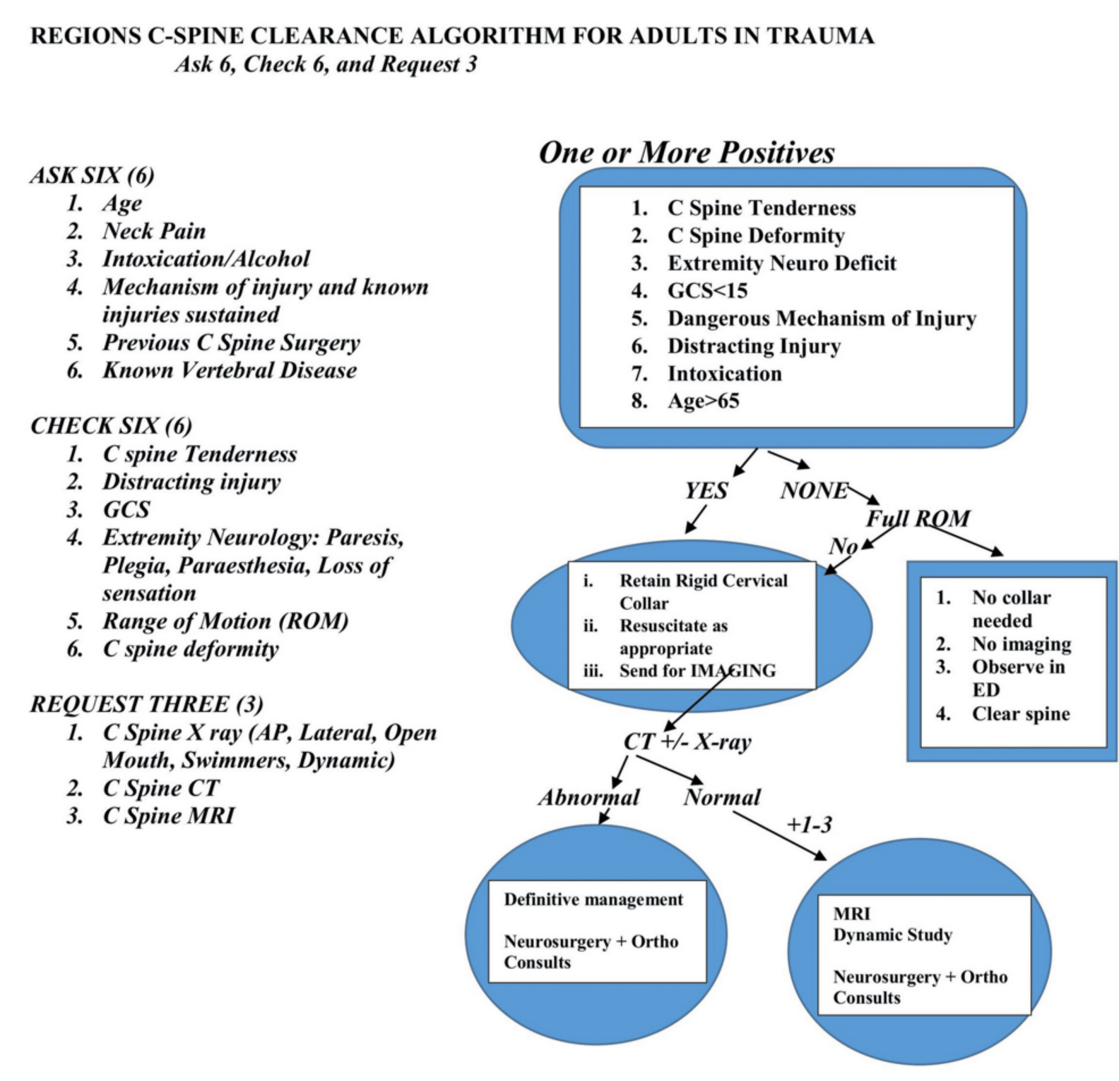
Initial concept C Spine: Cervical Spine, GCS: Glasgow Coma Score, ROM: Range of Motion, AP: Anteroposterior, CT: Computed Tomography, MRI: Magnetic Resonance Imaging, ED: Emergency Department, Ortho: Spinal Orthopedic Surgeons The image is an adaptation of the Canadian C-spine rule [6] and National Emergency X-Radiography Utilization Study (NEXUS) Criteria [7], developed by the author Ajah HE. Glasgow Coma Scale [10].

**Figure 2:**
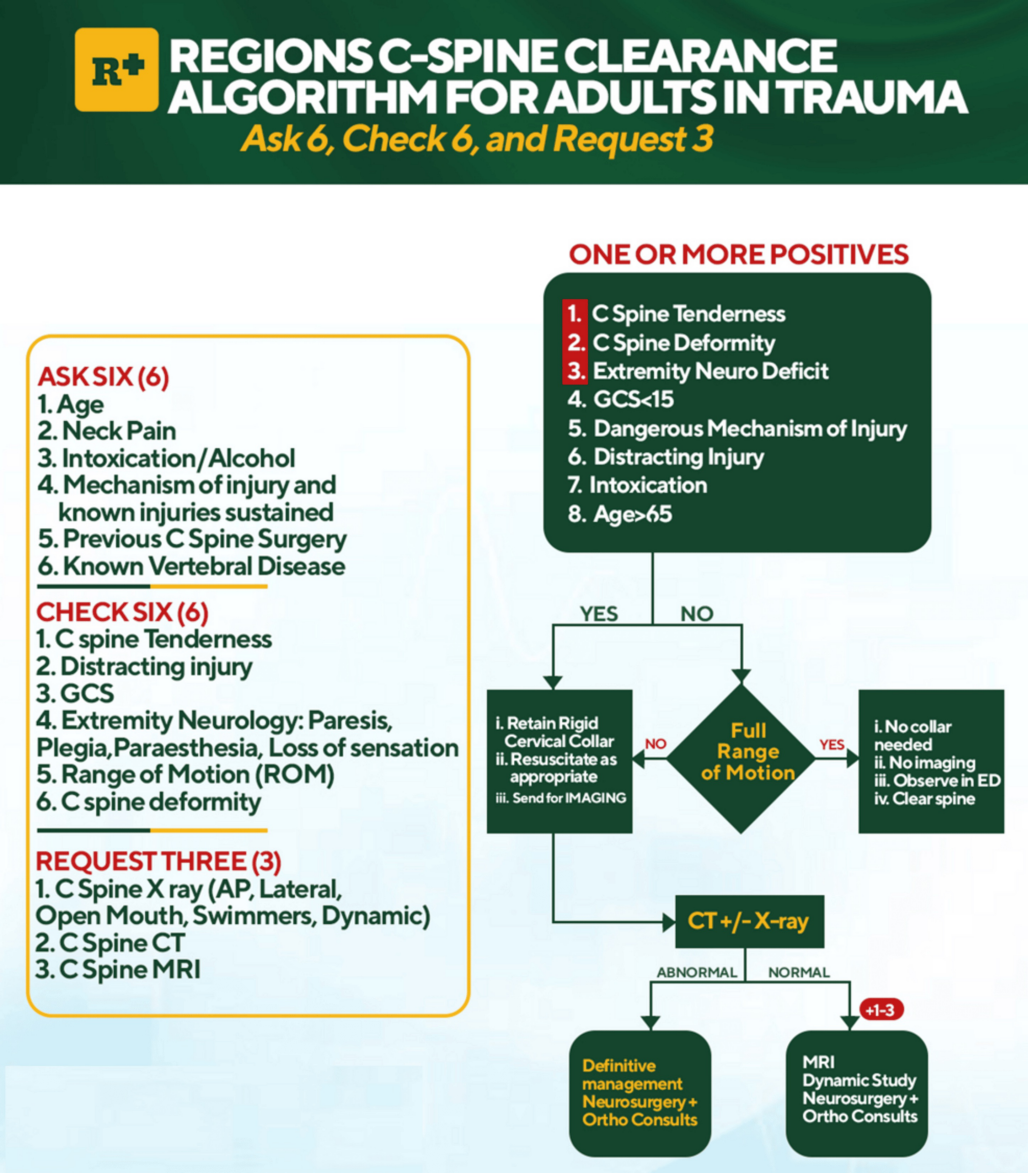
Final design C Spine: Cervical Spine, GCS: Glasgow Coma Score, ROM: Range of Motion, AP: Anteroposterior, CT: Computed Tomography, MRI: Magnetic Resonance Imaging, ED: Emergency Department, Ortho: Spinal Orthopedic Surgeons The image is an adaptation of the Canadian C-spine rule [6] and National Emergency X-Radiography Utilization Study (NEXUS) Criteria [7], developed by the author Ajah HE. Glasgow Coma Scale [10].

The hybrid protocol (Figure 1), which integrates key elements of the NEXUS Criteria and Canadian C-spine Rule, was developed to encompass all aspects of the C-spine assessment needed to make a specialist/imaging referral or clear the cervical spine. The Algorithm follows the Ask six, Check six, and Request three framework to emphasize the comprehensive information needed by the emergency department clinician to make a decision. Ask six (Age, Neck Pain, Intoxication/Alcohol, Mechanism of Injury, Previous Cervical Spine Surgery, Known Vertebral Disease), Check six (Cervical spine tenderness, Distracting Injury, Glasgow Coma Score, Extremity Neurology, Range of Motion, Cervical Spine Deformity), Request three (Cervical Spine X-ray, Computed Tomography and/or Magnetic Resonance Imaging). These items, representing the information the clinician should aim to have, are listed on the left of the design (Figure 2), whereas the flowchart to the right aims to direct the clinician on how to safely obtain and use this information.

The local guideline highlights eight high-risk factors that warrant radiological imaging: Cervical Spine Tenderness, Cervical Spine Deformity, Extremity Neurologic Deficit, Glasgow Coma Score less than 15, Dangerous Mechanism of Injury, Distracting Injury, Intoxication, and Age > 65 years. Patients with none of these can have a range of motion assessment, and the C-spine can be safely cleared if they demonstrate a full range of motion (up to 45-degree rotation following the Canadian C-Spine Rule). It further clarifies the need for further imaging, like an MRI and specialist consultation for the suspected C-spine injured patient with negative CT imaging and persisting Cervical Spine Tenderness, Deformity, or Extremity Neurology. In a single glance, the clinician can review the required history, clinical examination, and available investigations required to resolve the cervical spine in trauma. The protocol was adapted to local clinical workflows and displayed prominently in the emergency department and the intensive care unit.

Education, implementation, and preliminary assessment

The initial education was on the NEXUS criteria, the Canadian C-spine Rule, and general revision of the primary survey principles in trauma care. Then the hybrid locally adapted algorithm was introduced via a series of didactic lectures and hands-on training sessions involving the hospital’s Trauma/Emergency Department Team, Rapid Response Team members, and relevant specialists, including the attending neurosurgeons. Visual aids in the form of posters and digital materials were placed in high-traffic clinical areas, including the emergency department and the intensive care unit. In the preliminary survey following this, 100% (n=12) of clinicians reported greater confidence in clearing the C-spine, against 8.3% pre-training on the hybrid algorithm. There was particularly positive feedback on the reduction in the anxiety that comes with dealing with a new trauma patient with possible cervical spine injury, as decisions become mainly standardized, and every specialty attending trauma calls is guided by a unified protocol.

## Discussion

When the presence of cervical spine injury is unclear, the safest course of action is immobilization. However, continuing immobilization for longer than necessary carries both immediate and long-term adverse risks to the patient, including discomfort, pressure injury, elevated intracranial pressure and venous obstruction, difficult intubation and loss of airway, thromboembolism, challenge with oral care and enteral feeding [5]. The availability of international evidence-based decision-making tools to resolve cervical spine trauma has proven useful but not without limitation. The NEXUS criteria are reliable in a broad trauma population but less so in older patients, whereas the Canadian C-spine Rule requires a cooperative and stable patient. The locally adapted hybrid algorithm integrates elements from these two tools to ensure applicability across a wider patient population and singularity of principle in a local low-volume trauma unit. It further incorporates all aspects of the holistic cervical spine assessment required in an emergency setting into a three-part 15-point memorable item: Ask six, Check six, Request three. It is designed to be easily utilized by clinicians of all levels and ensure uniformity of communication within the local unit. 

Only about 2% of cervical spine injuries in trauma are clinically important, yet a missed diagnosis can be catastrophic [11]. Identifying or ruling this out is the goal of clinicians aiming to clear the cervical spine of a patient in trauma. There is a constant need to balance avoiding unnecessary imaging with avoiding missed injuries. Clinical decision-making tools like the NEXUS criteria and Canadian C-spine rules exist to aid clinicians in making this distinction. As these tools are not particularly adopted as national guidelines, it is good practice for trauma units to have a written cervical spine clearance guideline to unify language and enhance the efficient assessment of the trauma patient with suspected cervical spine injury.

Developing a decision-making tool like the Canadian C-spine rule takes a series of extensive, high-quality prospective multi-center studies. While the current decision-making tools have their limitations, they are sufficiently sensitive and the product of high-quality research to retain utility across trauma units. Thus, the hybrid algorithm we have developed strengthens the utility of these decision-making tools while aiming to provide a more comprehensive assessment aid for emergency department clinicians. Ongoing efforts are geared towards collecting long-term comprehensive pre- and post-longitudinal data to evaluate its impact on missed cervical spine injury in the facility, pending relevant approvals for the study. This will come on the heels of wider adoption and possible integration of the algorithm into the facility’s electronic medical software.

## Conclusions

The implementation of a hybrid cervical spine clearance algorithm in a local low-volume trauma unit has led to increased clinician confidence and improved adherence to a structured assessment of the cervical spine in trauma. The tool aims to simplify decision-making for emergency department clinicians, regardless of level of training. It currently applies to a low-volume trauma unit with limited human resources in trauma care but has the potential for broader regional and national implementation to improve trauma care and outcomes.

Further validation will rely on ongoing audits to provide evidence of effectiveness in reducing missed cervical spine injuries, detecting clinically important cervical spine injuries, and long-term physician adherence and satisfaction with the tool. The algorithm integrates elements of existing high-quality decision-making tools with additional details to make a robust, immediate cervical spine assessment in the emergency department.
